# Spirulina Liquid Extract Protects against Fibrosis Related to Non-Alcoholic Steatohepatitis and Increases Ursodeoxycholic Acid

**DOI:** 10.3390/nu11010194

**Published:** 2019-01-18

**Authors:** Marine Coué, Angela Tesse, Juliette Falewée, Audrey Aguesse, Mikaël Croyal, Lionel Fizanne, Julien Chaigneau, Jérôme Boursier, Khadija Ouguerram

**Affiliations:** 1 UMR 1280 Physiopathologie des Adaptations Nutritionnelles (PhAN), INRA, Université de Nantes, 44 093 Nantes, France; marine.coue@univ-nantes.fr (M.C.); juliette.falewee@outlook.com (J.F.); Audrey.Aguesse@univ-nantes.fr (A.A.); Mikael.Croyal@univ-nantes.fr (M.C.); 2 Centre de Recherche en Nutrition Humaine Ouest (CRNHO), 44 093 Nantes, France; 3 UMR 1087 Institut du Thorax, Université de Nantes, 44 007 Nantes, France; Angela.Tesse@univ-nantes.fr; 4 EA 3859, Hémodynamique, Interaction Fibrose et Invasivité Tumorales Hépatiques (HIFIH), 49 933 Angers, France; lionel.fizanne@univ-angers.fr (L.F.); julien.chaigneau@univ-angers.fr (J.C.); JeBoursier@chu-angers.fr (J.B.)

**Keywords:** spirulina liquid extract, non-alcoholic steatohepatitis, inflammation, oxidative stress, fibrosis, bile acids

## Abstract

Non-alcoholic steatohepatitis (NASH) is characterized by an excess of lipids and oxidative stress in the liver. Spirulina was reported to possess hypolipemic and antioxidative effects and might counteract NASH development. C57Bl/6J mice were fed a western diet (WD) during 25 weeks with or without spirulina liquid extract (SLE) at 2 different doses (WDS1 and WDS2 groups) in drinking water. Liver histology, inflammation, and oxidative stress were assessed as well as glucose tolerance status, lipid metabolism, and gallbladder bile acid profile. WDS2 gained significantly less weight than WD. Liver weight-to-body weight ratio and plasma alanine aminotransferase were significantly lower in WDS2 mice. A reduced liver fibrosis and NFκBp65 protein expression were measured in the supplemented group as a lower accumulation of superoxide anion, nitric oxide, and thiobarbituric reactive substances. WDS2 mice showed also a preserved glucose tolerance, a strong decrease of plasma cholesterol, and a significant increase of gallbladder ursodeoxycholic acid and β-muricholic acid. Our findings demonstrate a protective effect of SLE against WD induced NASH that is related to less inflammation and oxidative stress, a preserved glucose tolerance, and less hepatotoxic bile acid profile.

## 1. Introduction

Non-alcoholic fatty liver disease (NAFLD) encompasses a spectrum of diseases, including simple steatosis and non-alcoholic steatohepatitis (NASH), which may lead to cirrhosis and hepatocellular carcinoma. NASH reflects installation of inflammation and frequently progresses to fibrosis. It was published that fibrosis stage increases the risk of liver-related mortality exponentially [1]. By 2020, NASH is projected to be the major indication for liver transplantation [2]. Major risk factors include obesity, insulin resistance (IR), and type-2 diabetes (T2D) [3].

Although some drugs are testing in clinical trials [4], no approved medication exists for NASH treatment for now. NAFLD management for patients consists of the adoption of a healthy lifestyle involving weight loss and physical activity [5]. Nutrition can help prevent NAFLD occurring. Among nutraceutical agents widely used, *Arthrospira platensis*, commonly named Spirulina, has been consumed since antiquity in Aztec civilizations. Spirulina is a cyanobacteria capable of photosynthetic activity and this implies an organism rich in antioxidants [6]. Most of spirulina antioxidant activities are proved to be related to C-phycocyanin (C-PC) activity [7], but other molecules such as minerals can induce additive or even synergic beneficial effects [8,9].

Underlying mechanisms of NAFLD development remain poorly understood, but several factors are known to be involved in NASH progression, such as inflammation, oxidative stress, insulin resistance, lipotoxicity, and bile acid toxicity [10,11,12]. Preclinical and clinical randomized controlled trials already put forward hypolipidemic, antioxidant, glucose lowering and anti-inflammatory Spirulina properties, as already reviewed [6,13,14]. Furthermore, Pak et al. published that NASH gravity can be alleviated with the supplementation of spirulina or C-PC in rats under a choline-deficient diet [15]. So far, no study on spirulina/C-PC supplementation has analyzed all NASH risk factors together. Moreover, no study was reported on the effect of Spirulina on bile acid metabolism.

The purpose of the current research was to determine whether spirulina liquid extract (SLE) supplementation, a concentrate of C-PC, can protect against NASH development and its potential underlying mechanisms in mice under a western diet. Mouse model was preferred over rat because it allows analysis of gallbladder bile acids. Our results suggest a protection of hepatic fibrosis through preservation against inflammation, oxidative stress, and whole body insulin resistance with SLE consumption associated with an increased gallbladder ursodeoxycholic acid (UDCA) concentration.

## 2. Materials and Methods

### 2.1. Spirulina Liquid Extract

SLE is a patented water extract of *Arthrospira platensis* (Number of patent: 17 52452). SLE contains a high quantity of C-PC and corresponds to the alga cytoplasmic hydrophilic compounds, meaning no insoluble fibers and no fat-soluble elements. It was obtained from AlgoSource Technologies, 44 600 Saint Nazaire, France. The biomass is cultivated in raceway at Assérac, France and the formulation is made in Guérande, France. The standardization of SLE was made on C-PC content. SLE composition is presented in Table S1.

### 2.2. Mice and Diets

Five-week-old C57Bl/6J male mice (Janvier labs, Le Genest-Saint-Isle, France) were housed 5 animals/cage with *ad libitum* access to water and food and with constant 12/12 h light/dark cycles. Mice were fed for 25 weeks since their seventh week of age with a chow diet (A04; Safe Diets, Augy, France) (control group) or a western diet (WD): 45% of energy from fat enriched with cholesterol (2%) (U8958v250, Safe Diets) and fructose (42 g/L) (61252, Safe Diets) in drinking water. Composition of diets is available in Table S2. Supplemented mice received SLE at 60 and 300 mg C-PC/kg body weight/day (WDS1 and WDS2 groups respectively) in drinking water. All experimental procedures were approved by the Committee of Ethics in Animal Experiments of Pays de la Loire, France (Project No. APAFIS#6697) and performed according to the European Union regulations for the care and use of animals for experimental procedures (2010/63/EU).

### 2.3. Blood Analyses and Tissue Collection

At 12 h 00 am, after 4 h of fast, mice were anesthetized under isoflurane (5 L/min, 2–3%) and blood was collected by cardiac puncture into tubes containing EDTA K2 (Sarstedt, Marnay, France). Organs were rapidly excised and snap frozen in liquid nitrogen before being stored at −80 °C. Aspartate aminotransferase (ASAT), alanine aminotransferase (ALAT) were determined by photometry using standard clinical biochemical procedures (Roche Diagnostics, Meylan, France). Fasting plasma total cholesterol (TC), triglycerides (TG) and non-esterified fatty acids (NEFA) were performed using enzymatic assay (DiaSys, Grabels, France). Isolation of lipoproteins was performed by fast protein liquid chromatography (ÄKTAFPLC) (GE Healthcare, Buc, France) using 200µL of plasma [16]. Plasma apolipoproteins (ApoA-I, ApoB100, ApoC-II, ApoC-III, and ApoE) were measured by liquid chromatography–tandem mass spectrometry (LC-MS/MS) as previously described [17] with slight modifications due to specific mouse isoforms (Table S3). Gallbladder bile acids (BA) were further analyzed by LC-MS/MS as recently published [18]. Thiobarbituric acid reactive substance (TBARS) content in liver were quantified using the fluorimetric procedure of Yagi [19].

### 2.4. Liver Steatosis and Fibrosis Quantification

Briefly, a slice of each liver lobe 1 and 2 were fixed in 4% paraformaldehyde for 24 h before paraffin-embedding. Then 5 µm thick sections were stained with hematoxylin-eosin-saffron (HES) or 0.1% picrosirius red (area of steatosis and fibrosis) solution. The entire stained specimen was analyzed by an automatic thresholding technique using an algorithm developed in HIFIH laboratory (EA 3859, Angers, France) as previously described [20].

### 2.5. Western Blot

Livers were homogenized in RIPA lysis buffer (EMD Millipore Corp, Temecula, CA, USA), containing 10 µL/mL of phosphatase I and II inhibitor and protease inhibitor (Sigma-Aldrich, St. Quentin Fallavier, France). Tissue homogenates were centrifuged for 15 min at 10,000 g, and supernatants were stored at −80 °C. Equal amount of proteins (40 µg) were run on a 4–15% gradient Mini-protean TGX gels (Bio-Rad, Marnes-la-Coquette, France), transferred onto Trans-Blot Turbo nitrocellulose membranes (Bio-Rad, Marnes-la-Coquette, France) and blotted with the following primary antibodies: phospho-IκBα (1/500), total-IκBα (1/500), Nuclear Factor kappa-B (NFκB)p65 (1/200) (all Santa Cruz Biotechnology, Heidelberg, Germany) and β-actin (1/5000, Sigma-Aldrich, St. Quentin Fallavier, France). Anti-mouse, anti-goat, or anti-rabbit IgG labeled with Dylight 800 or Dylight 680 was used as secondary antibodies. Proteins were determined by Infrared fluorescent detection (Odyssey, LI-COR Biosciences, Boulogne-Billancourt, France) and quantified using Image Studio Lab v5.2 software (LI-COR Biosciences, Boulogne-Billancourt, France).

### 2.6. Superoxide Anion (O_2_^−^) and Nitric Oxide (NO) Measurements by Electronic Paramagnetic Resonance (EPR)

As published previously [21], a piece of liver was used for O_2_^−^ detection and incubated in a Krebs-Hepes solution containing 1-hydroxy-3 methoxycarbonyl-2,2,5,5-tetramethylpyrrolidin (CMH, 500 mM, Noxygen; Denzlingen, Germany) as spin probe, deferoxamin (25 mM, Sigma-Aldrich, St. Quentin Fallavier, France) and diethyldithiocarbamate (DETC, 5 mM, Sigma-Aldrich, St. Quentin Fallavier, France) at 37 °C for 45 min. Then, each sample was frozen in liquid nitrogen and analyzed in a Dewar flask at 77 °K by EPR using a table-top x-band spectrometer Miniscope (MS5000, Freiberg Instruments, Freiberg, Germany). Instrument settings were 10 mW of microwave power, 0.400 mT of amplitude modulation, 100 kHz of modulation frequency, 60 s of sweep time and 3 scans. 

For NO measurements another piece of the same liver lobe was incubated for 45 min at 37 °C in a pale yellow-brown opalescent colloid Fe-(DETC)2 solution as spin trap. The colloid was obtained by separately dissolving NaDETC (3.6 mg, Sigma-Aldrich) or FeSO_4_-7H_2_O (2.3 mg, Sigma Aldrich St. Quentin Fallavier, France) in 10 mL of ice-cold Krebs-Hepes buffer under nitrogen gas bubbling and mixing the two solutions immediately. Then, samples were frozen in liquid nitrogen and analyzed by EPR Miniscope MS5000. Recordings were made at 77 °K in a Dewar flask. The instrument settings were 10 mW of microwave power, 1 mT of amplitude modulation, 100 kHz of modulation frequency, 150 s of sweep time and 3 scans. 

Signals were quantified by measuring amplitude peaks of the spectra after baseline correction. All values were expressed in arbitrary units (a.u.)/g of tissue proteins.

### 2.7. Liver Staining and Confocal Microscopy Imaging

Frozen sections of liver (10 μm thick) on glass slides were used for the in situ detection of O_2_^−^ by incubation with the oxidative fluorescent dye dihydroethidine (DHE, Sigma-Aldrich, St. Quentin Fallavier, France) which oxidizes to ethidium bromide in presence of O_2_^−^ showing a red fluorescence. Digital image recording was performed using the NIS element software. Images were analyzed and processed by Fiji software.

### 2.8. Glucose Tolerance Test

Four hour-fasted mice were injected intraperitoneally at 2h00 pm with a bolus of D-glucose at 2 g/kg (Sigma-Aldrich, St. Quentin Fallavier, France) for glucose tolerance tests (GTT). Glycemia was monitored with a tail blood drop with a glucometer (Roche Diagnostics, Meylan, France) at 0, 15, 30, 45, 60, 90, and 120 min after injection. At the glycemic peak, 15 min after D-glucose injection, blood was harvested in tubes containing EDTA (Sarstedt, Marnay, Germany). 10 µL of plasma were then isolated after centrifugation (15 min, 5000 g, 4 °C) to measure insulin using an ELISA kit (Alpco, Salem, NH, USA).

### 2.9. Real-Time qRT-PCR

Total tissue RNA extraction was processed using TRizol reagent (Life Technologies, Saint Aubin, France) according to manufacturer instructions. After reverse transcription of 1 µg total RNA realized with SuperScript III Reverse Transcriptase (Life Technologies, Saint Aubin, France) and DNAse treatment (Promega, Charbonnières-les-Bains, France), samples were analyzed on a Bio-Rad CFX Manager system (Bio-Rad, Marnes-la-Coquette, France). All primer sequences are presented in Table S4 (Eurofins, Nantes, France). Expression data were normalized by the 2^(ΔCt)^ method using *Tata-box binding protein* (*Tbp*) as internal control.

### 2.10. Statistics

Experimental values were presented as the mean ± SEM (standard error to the mean). Statistical analyses were performed using one-way analysis of variance (ANOVA) with Holm–Sidak’s multiple comparison test. Kruskal–Wallis with Dunn’s multiple comparison test was applied to variables not meeting normality and homoscedasticity assumptions. Correlations were analyzed with Spearman correlation. *p*-values lower than 0.05 were considered significant. All analyses were performed with GraphPad Prism 6 software (GraphPad Software, San Diego, CA, USA).

## 3. Results

### 3.1. Spirulina Alleviates Diet Induced Body Weight Gain

**Figure 1:**
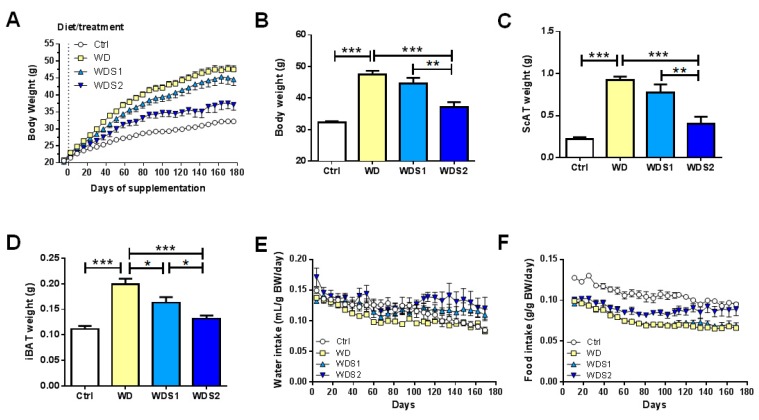
Spirulina liquid extract protects against diet induced obesity and increases food intake. (**A**) Follow up of body weight over 25 weeks and (**B**) final body weight. (**C**) Subcutaneous adipose tissue (ScAT) and (**D**) interscapular brown adipose tissue (iBAT) weight at the end of the supplementation. (**E**) Follow up of water intake and (**F**) food intake calculated as water or food intake-to-body weight ratio for each mouse and every week. * *p* < 0.05, ** *p* < 0.01, *** *p* < 0.001 (*n* = 9–10/group), ANOVA.

Mice under WD since seven weeks of age gained significantly more weight than the control group and WDS2 showed a decrease in body weight compared to WD (−21%, *p* < 0.001) (Ctrl: 32.2 g ± 0.4; WD: 47.5 g ± 1.1; WDS1: 44.6 g ± 1.8 and WDS2: 37.6 g ± 1.5) (Figure 1A,B). These results were associated with a decreased weight of subcutaneous (ScAT) and interscapular brown fat (iBAT) depots in WDS2 mice by 56% and 24% respectively (Figure 1C,D). No significant difference in beverage consumption was observed between these four groups when reported to their body weight (Figure 1E and Figure S1A). WD mice showed a statistical reduced food intake in comparison to control mice when reported to their body weight (−30%, *p* < 0.001) (Figure 1F and Figure S1B). WDS2 group ate significantly more than WD and WDS1 mice when normalized to their body weight (+21%, *p* < 0.01) (Figure 1F and Figure S1B). Together, these results suggest that SLE alleviates diet induced obesity despite a greater food intake.

### 3.2. NASH and Liver Fibrosis is Prevented with Spirulina Supplementation

**Figure 2:**
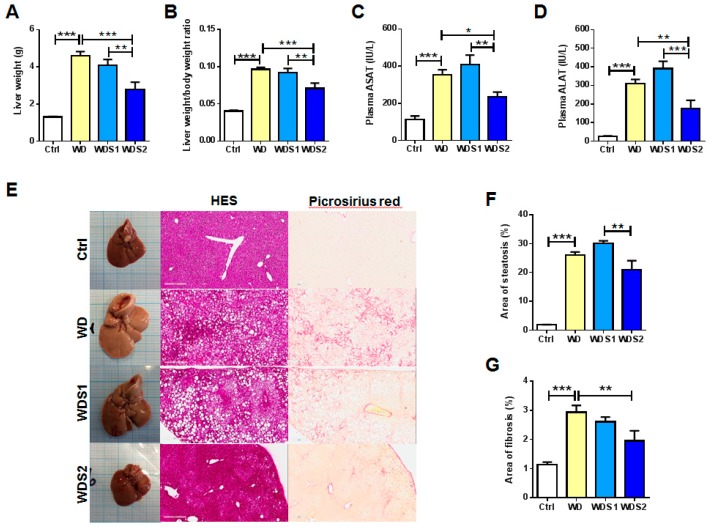
Spirulina alleviates western diet induced hepatic fibrosis and inflammation. (**A**) Liver weight, (**B**) liver weight to body weight ratio, (**C**) plasma aspartate aminotransferase (ASAT) and (**D**) plasma alanine aminotransferase (ALAT) measured at the end of the protocol. (**E**) Whole liver pictures, hematoxylin-eosin-saffron (HES) (Bar = 300 µm) and picrosirius red (Bar = 200 µm) staining images allowing quantification of (**F**) area of steatosis and (**G**) area of fibrosis. * *p* < 0.05, ** *p* < 0.01, *** *p* < 0.001 (*n* = 9–10/group), ANOVA.

The investigation of mouse liver health indicated that WD mice had a significant bigger liver weight and liver weight-to-body weight ratio compared to control mice (Figure 2A,B). Compared to WD mice, these two parameters were reduced in WDS2 group. Higher ASAT and ALAT levels were measured in plasma of WD mice than their control counterpart reflecting hepatocyte damages (Figure 2C,D). WDS2 mice presented significant lower concentrations of plasma ASAT and ALAT compared to WD group (Figure 2C,D). Figure 2E shows whole liver images and cross sections after HES or picrosirius red staining which have served for the quantification of steatosis and fibrosis. Hepatic steatosis area revealed a strong elevation in WD mice by more than 14 fold that was not statistically alleviated in SLE supplemented mice even if WDS2 mice expressed significantly less fibrosis than WDS1 (Ctrl: 1.82% ± 0.08; WD: 26.04% ± 1.07; WDS1: 30.09% ± 0.94 and WDS2: 20.99% ± 3.14) (Figure 2F). Fibrosis raised almost 3 fold in WD group and dropped significantly in WDS2 mice (Ctrl: 1.13% ± 0.09; WD: 2.94% ± 0.23; WDS1: 2.61% ± 0.17 and WDS2: 1.96% ± 0.34) (Figure 2G). 

Some key hepatic metabolic genes were analyzed and reported in Table 1. *Slc2a2* gene was downregulated in WDS2 in comparison to WD (−34%, *p* < 0.05) as *Cd36* (−43%, *p* < 0.05). These mice also showed a lower expression of *Acetyl-CoA carboxylase-1* (*Acc1*, −38%, *p* < 0.05), *Stearoyl-CoA desaturase-1* (*Scd-1*, −42%, *p* < 0.01), *Sterol regulatory element-binding protein-1* (*Srebp-1*, −25%, *p* = 0.06), and *Diacylglycerol O-acyltransferase-1* (*Dgat-1*, −21%, *p* < 0.05) as lipogenic genes. In this study, the transcription factor *Peroxisome proliferator-activated receptor α* (*Ppar-α*) and the mitochondrial acyl-CoA transfer gene *Carnitine palmitoyltransferase1-a* (*Cpt1-a*) involved in lipid oxidation were significantly lessening in WDS2 rodents. Finally, livers from the highest dose of SLE supplementation expressed less mRNA of *Collagen type 1 α1* (*Col1a1*, −48%, *p* = 0.01), *Tissue inhibitor of metalloproteinase 1* (*Timp1*, −50%, *p* < 0.05), *Transforming growth factor β1* (*Tgf-*β1, −31%, *p* < 0.05), and *Toll-like receptor 9* (*Tlr9*, −50%, *p* < 0.001) implied in fibrogenesis and/or inflammation. Globally, SLE partially prevents WD induced hepatic inflammation and fibrosis installation in mice.

### 3.3. Liver from Spirulina Supplemented Mice Are Protected against Reactive Oxygen and nitrogen Species Accumulation Related to WD

**Figure 3:**
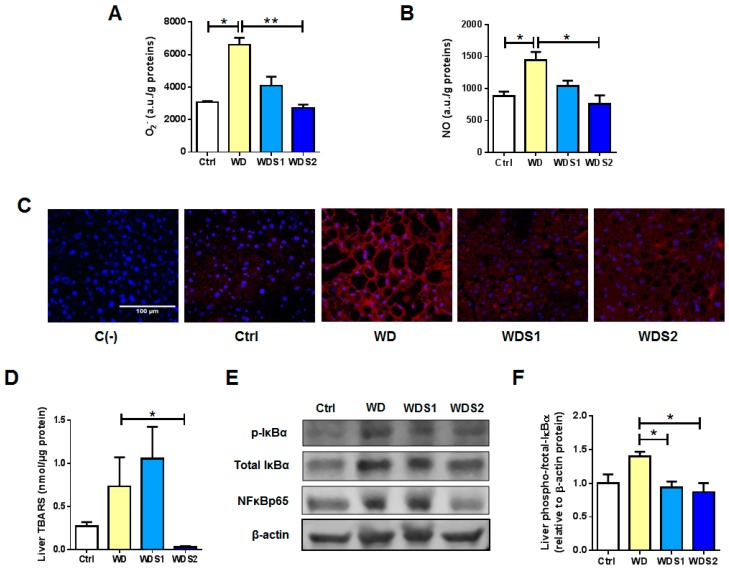
Spirulina liquid extract inhibits western diet induced oxidative stress and inflammation. Hepatic (**A**) superoxide anion (O_2_^−^) and (**B**) nitric oxide (NO) accumulation where quantified the day of sacrifice. (**C**) Confocal images of O_2_^−^ production in the liver detected by dihydroethidium staining in red fluorescence. Cell nuclei were visualized by DAPI in blue. C(-) negative control without dihydroethidine (DHE). Bar = 100 µm. (**D**) Quantification of hepatic thiobarbituric acid reactive substances (TBARS). (**E**) Representative blots of phospho- and total-IκBα, NFκBp65 and β-actin proteins in liver. (**F**) Quantitative bar graph of liver phospho- to total-IκBα ratio. * *p* < 0.05, ** *p* < 0.01 (*n* = 4–5/group), Kruskal–Wallis.

Liver O_2_^−^ and NO contents were significantly increased in WD group by 115 and 63% respectively (Figure 3A,B) *versus* control. Hepatic level of O_2_^−^ and NO in WDS2 mice were significantly reduced by 59 and 47% respectively, to levels similar to control mice (Figure 3A,B). Moreover, histochemical DHE staining evidenced an increased O_2_^−^ production in WD livers compared to very weak staining detected in control mice while WDS1 and WDS2 groups exhibited an intermediate state (Figure 3C). Hepatic TBARS production, as a marker of lipid peroxidation, was also significantly decreased by 96% in WDS2 group (Figure 3D). Hepatic anti-oxidative gene expression, including *Superoxide dismutase 1 (Sod1)* and *Catalase*, were significantly decreased in WD compared to Ctrl group contrary to *Glutathione peroxidase (Gpx)* (Figure S2A–C). Solely, *Sod1* expression was decreased in WDS2 mice. These gene expressions were not modified in other oxidative tissues such as skeletal muscle (SkM) and iBAT in WDS2 *versus* WD mice (Figure S2D–I). Overall, liver oxidative stress status seems to be relieved in SLE supplemented mice. Representative western blots show that activated phospho-IκBα and its target NFκBp65 were less present in liver of WDS2 compared to WD mice (Figure 3E). Figure 3F represents quantitative phospho- to total-IκBα ratio that was significantly weaker in WDS1 and WDS2 livers.

### 3.4. WD Induced Insulin Resistance Is Reduced with SLE Supplementation

**Figure 4:**
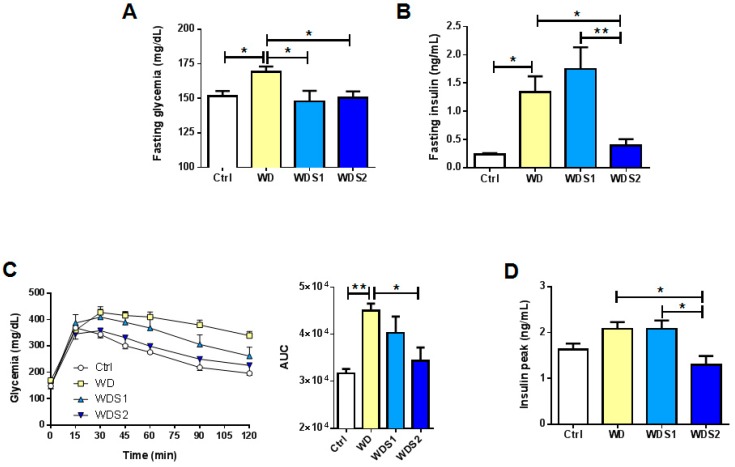
Spirulina supplementation preserves insulin sensitivity. (**A**) Four hours fasting blood glucose and (**B**) insulin at the end of the protocol. (**C**) Time course of blood glucose levels during an intraperitoneal glucose tolerance test (ipGTT) (2 g/kg body weight) performed after 24 weeks of spirulina liquid extract (SLE) administration and corresponding area under the curve (AUC). (**D**) Plasma insulin levels 15 min after bolus glucose injection during ipGTT. * *p* < 0.05, ** *p* < 0.01 (*n* = 9–10/group), ANOVA.

Since IR is a key factor in the initiation and progression of NAFLD, we assessed the effect of SLE on glucose tolerance. As expected, WD mice presented an impaired fasting glycemia compared to control, whereas both concentrations of SLE supplementation significantly reduced fasting glycemia to the same level as control mice (Ctrl: 148 mg/dL ± 1.9; WD: 169 mg/dL ± 3.7; WDS1: 148 mg/dL ± 7.7 and WDS2: 153 mg/dL ± 4.6) (Figure 4A). Fasting insulinemia increased significantly in WD and was statistically alleviated in WDS2 mice (Figure 4B). We measured a significant improvement of glucose tolerance in WDS2 mice as measured by the glucose tolerance test (GTT) and area under the curve calculation (Figure 4C). We also quantified insulinemia 15 min after the injection of glucose bolus during GTT in each group. Insulin was statistically lower in WDS2 mice than WD (Figure 4D). Collectively, these findings indicate a protective role of SLE against WD induced IR.

### 3.5. SLE Maintains a Normal Plasma Lipid Profile as a Marker of Liver Functionality

**Figure 5:**
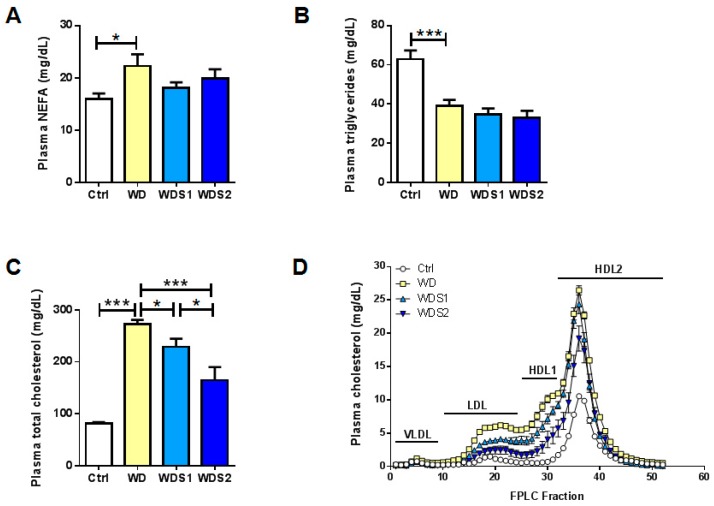
Spirulina administration prevents western diet induced hypercholesterolemia. (**A**) Quantification of plasma non-esterified fatty acids (NEFA) and (**B**) triglycerides. (**C**) Plasma total cholesterol level and (**D**) cholesterol repartition among very low density lipoprotein (VLDL), low density lipoprotein (LDL) and high density lipoprotein (HDL). * *p* < 0.05, *** *p* < 0.001 (*n* = 9–10/group), ANOVA.

To study mice liver functionality, fasting plasma lipids were investigated. NEFA were statistically enhanced solely in WD compared to Ctrl mice (Figure 5A). A strong lessening of plasma TG was observed in WD compared to Ctrl mice and no change was found in SLE supplemented groups (Figure 5B). Gene expression of *Microsomal TG transfer protein* (*Mttp*) was unchanged between conditions in liver (Figure S3A) whereas *Lipoprotein lipase* (*Lpl*) was decreased significantly in ScAT and *Cd36* was significantly lower in iBAT and ScAT in WDS2 mice compared to control mice (Figure S3B,C respectively). Plasma cholesterol in WD mice was dramatically increased by 3 fold, compared to control mice (Figure 5C). WDS1 and WDS2 mice presented a significant decrease in cholesterolemia in comparison with WD (Ctrl: 81.2 mg/dL ± 2.9; WD: 272.2 mg/dL ± 8.6; WDS1: 228.8 mg/dL ± 16 and WDS2: 165.0 mg/dL ± 25.3). Cholesterol carried by LDL, HDL1 and HDL2 rose sorely in WD mice, but not VLDL-cholesterol, as shown by FPLC graph and the sum of cholesterol concentration for each lipoprotein (Figure 5D, Table 2). All lipoprotein cholesterol contents were dose dependently lesser with the augmentation of SLE dose. WD also strongly downregulated hepatic *hydroxyl-methyl-glutaryl CoA reductase* (*Hmg-CoA R)* expression in WDS1 and WDS2 mice compared to control mice (Figure S3D).

Major plasma apolipoproteins A-I, B100, E, C-II, and C-III were also quantified (Table 2). All of them were dramatically elevated in WD fed animals compared to control and significantly decreased after high dose of SLE supplementation. On the whole, these elements provide evidence for less cholesterol accumulation in plasma from mice receiving SLE.

### 3.6. SLE Promotes a Less Hydrophobic Bile Acid Profile and Increased Ursodeoxycholic Acid Concentration

**Figure 6:**
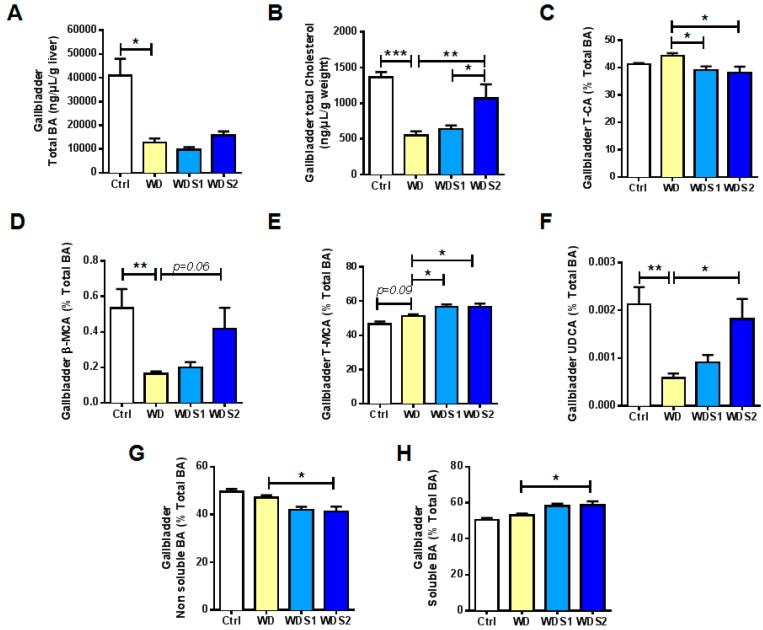
Spirulina liquid extract reduces hydrophobic BA and enhances ursodeoxycholic acid content. Gallbladder (**A**) total BA, (**B**) cholesterol, (**C**) tauro-cholic acid (T-CA), (**D**) β-muricholic acid (MCA), (**E**) tauro-total-MCA and (**F**) ursodeoxycholic acid (UDCA) content. Gallbladder (**G**) soluble BA calculated as the sum of unconjugated-tauro-glyco-UDCA and MCA and (**H**) non soluble BA calculated as the sum of unconjugated-tauro-glyco-CA, deoxycholic acid (DCA), chenodeoxycholic acid and lithocholic acid. * *p* < 0.05, ** *p* < 0.01, *** *p* < 0.001 vs. WD (*n* = 9–10/group), ANOVA.

Another important role of the liver is the management of lipids through BA metabolism. In the present study, WD has severely impacted gallbladder total BA production and total cholesterol excreted in the gallbladder compared to control (Figure 6A,B). If there was no difference of gallbladder total BA content in WD and WDS2 mice (Figure 6A), bile total cholesterol content has been doubled in WDS2 mice (Figure 6B). Hydrophobic taurocholic acid (T-CA) was significantly decreased in WDS1 and WDS2 compared to WD mice (Figure 6C). By contrast, hydrophilic β- and tauro-muricholic acid (MCA) were statistically enhanced in WDS2 (Figure 6D,E). Furthermore, WDS2 mice had a significant rise by 3 fold of hydrophilic ursodeoxycholic acid (UDCA) almost reaching control level (Figure 6F). These results led to a lower hydrophobic BA pool and a higher soluble BA pool in gallbladder of WDS2 mice (Figure 6G,H). Importantly, gallbladder UDCA content was observed to be inversely correlated to metabolic relevant parameters as fasting glycemia, liver weight-to-body weight ratio, hepatic O_2_^−^ content, plasma ALAT or whether fibrosis area (Table 3). It should be noted that those same variables were also inversely correlated with gallbladder β-MCA (Table S5). In summary, these results state for a less toxic BA pool after high dose of SLE administration.

## 4. Discussion

The present study analyzed hepatoprotective effects of 25 weeks of Spirulina liquid extract (SLE) supplementation that is a concentrate of C-PC, a powerful antioxidant, at doses of 60 and 300 mg/kg/day. These results provide some mechanistic explanations for SLE effects, involving for the first time the role on bile acids (BA) metabolism. SLE prevents WD induced obesity, hepatic fibrosis related to NASH, inflammation, reactive oxygen species (ROS), and insulin resistance (IR). All these parameters negatively correlate with gallbladder UDCA content that is significantly increased with the highest dose of SLE supplementation.

We first observed a protective effect of SLE on body weight and fat depot weight. A decrease of body mass index (BMI) in patients with hypertension receiving spirulina (2 g/day, 3 months) [22] and in supplemented NAFLD (6 g/day, 6 months) or obese patients (2 g/day, 12 weeks) has already been published [23,24]. Spirulina seems to be mainly effective in overweight/obese patients, while no effect on BMI was reported in normal weight, HIV-patients, or children with nephrotic syndrome [25,26]. The underlying mechanism of spirulina on BMI is still poorly understood. A study performed on a mouse metabolic syndrome model proposed that spirulina alleviates macrophage infiltration into visceral AT, preventing hepatic NEFA overflow and oxidative stress [27]. Another article realized on T2D KKA^y^ mice (100 mg C-PC/kg BW/day, 3 weeks) also showed a significant reduction of body weight [28]. In the present study, supplementation led to a lower *Scd1* mRNA expression. Indeed, SCD1-/- mice are described to be protected from high-fat diet-induced obesity related to down-regulation of lipogenic genes also measured in our study [29].

Surprisingly, WDS2 mice ate even more when normalized weekly food intake to their body weight. At first sight, this seems to be in contradiction with Zeinalian et al., showing that Spirulina supplementation at a dose of 1 g/day for 12 weeks was reported to effectively decreased appetite measured with visual analogue scale in obese individuals [30]. Another study revealed also a significant reduction of appetite score after a daily intake of 2 g of *Spirulina platensis* for 12 weeks [24]. This effect could be due to the high protein or fiber content of Spirulina. It is important to note that our results are associated with a decreased body weight gain over the 25 weeks. Higher food intake in WDS2 mice could be the consequence of higher energy expenditure and/or an elevated locomotor activity. Another possibility is that SLE inhibits nutrient absorption during digestion. This hypothesis is consistent with a previous research showing that *Spirulina platensis* concentrate significantly decreases micellar solubility in vitro [31].

In the present study, SLE supplementation alleviated WD induced NASH as reflected with less liver weight-to-body weight ratio, plasma ALAT, fibrosis, and inflammation. Our results are in agreement with data from a rat model of NASH receiving spirulina or C-PC [15]. In this article, both supplementations decreased plasma ASAT, ALAT, liver lipid peroxidation, ROS, and NFκBp65 nuclear import. Hepatic inflammation is a key contributor to the pathophysiology of NASH. Liver NFκB activation is a characteristic feature of human and animal model of NASH. We measured less phosphorylated Iκβ-to-total Iκβ ratio and NFκBp65 protein after SLE supplementation. It has been published that lipid and/or ROS accumulation induce inflammatory pathways by activating Iκβ kinase complex (composed of Iκκα, Iκκβ, and NFκB) [32].

In the current study, SLE showed no significant effect on hepatic steatosis. Clinically, simple liver steatosis is still considered “benign” but becomes alarming when inflammation and fibrosis develop as a signature of NASH. It has been published that accumulation of neutral lipids is a protective mechanism against NASH [33]. Importantly, the key point in NASH is not so much the quantity of liver lipids but the type of lipids accumulation and how they are packaged [34]. The improvement of fasting glycemia and inflammation in our supplemented mice probably suggest a reduced liver lipotoxicity.

Furthermore; our findings reveal a protection against WD induced O_2_^−^, inflammatory NO accumulation and lipid peroxidation marker in the liver of WDS2 mice validating antioxidant and anti-inflammatory properties of SLE. The reduction of NO levels in WDS2 livers could be related to decreased NFκBp65 activation because of its role in inducible NO synthase triggered expression during inflammatory process [35]. O_2_^−^ level effects could be due to ROS scavenging activity of SLE [36].

Moreover, accumulating evidence suggests that ROS play a major role in NAFLD initiation and progression [10,11]. This is associated with a lowering or no change in hepatic, skeletal muscle and interscapular brown fat anti-oxidative gene expression, meaning that SLE could act directly via C-PC and enable the saving of endogenous anti-oxidative defenses. Therefore, SLE could alleviate fibrosis development by its direct antioxidant effect and limit utilization of anti-oxidative enzymes, this restricts mitochondria over-oxidation of acetyl-CoA, as seen with the lower expression of *Ppar-α* and *Cpt1a*, and thus protect against fibrogenesis in WDS2 group, as shown with less *Tgf-β1*, *Col1a1* and *Timp1* mRNA. It was demonstrated that C-PC protects rat livers from cold ischemia/reperfusion injury, comprising decrease ALAT, ASAT, AP, and GPX activities [37]. The protective effect of SLE measured in our study is in accordance with previous published data obtained in T2D patients supplemented with Spirulina (8 g/day, 12 weeks) [38].

We then described a powerful protective effect of SLE on glucose tolerance state. This is in agreement with studies performed on 2 different mouse models of T2D supplemented with C-PC (100 and 200 mg/kg/day, 3–6 weeks) which strongly reduced fasting blood glucose, insulin, TG, and TC [28,39]. Furthermore, C-PC dose dependently decreased fasting glycemia in a T2D mouse model (50, 100, and 200 mg/kg, 4 weeks) [40]. This is accompanied by an increase in phosphorylation of hepatic IRS-1 tyrosine and Akt Ser473 in the liver and the pancreas. This was associated with greater hepatic and muscle glycogen accumulation, as well as pancreatic islet size reduction. 

Another result supporting the improvement of liver metabolic function is the modification of plasma cholesterol metabolism with SLE supplementation. We observed a decrease in plasma TC and in lipoprotein cholesterol in supplemented mice. While WD mice showed an increase of cholesterol and major apolipoproteins, SLE treated mice showed a C-PC dose dependent decrease of both TC and measured apolipoproteins. WD mice showed an appearance of large ApoE-rich HDL1 particles compared to control mice in a dose dependent manner. Elevated plasma HDL1 was previously reported in mice under high fat diet, in leptin-deficient and leptin-receptor-deficient mice [41,42]. SLE supplementation significantly decreased plasma HDL1. Lipid-lowering effect of spirulina was already reported in mice [27], rats [31], hamsters [7], rabbits [43], and humans [44,45]. Elevation of gallbladder cholesterol in our WDS2 mice could explain the lower plasma cholesterol concentration. Otherwise, Nagaoka et al. published that spirulina has in vitro the capacity to bind T-CA and decrease micellar solubility of cholesterol [31]. They showed that cholesterol absorption in Caco-2 cells was significantly reduced with micellar solution containing spirulina. They also fed rats with spirulina or C-PC, and both diets significantly lowered plasma cholesterol and enhanced fecal acid and steroids outputs [31].

We can also speculate that some protective effects of SLE may be mediated by UDCA. This is the first time according to our knowledge that a Spirulina product is proved to modulate BA metabolism and enhance UDCA concentration. Indeed, this hydrophilic BA is known to be less hepatotoxic than other BA, such as T-CA and T-DCA [46]. In the present study, T-CA dropped in WDS2 gallbladders, while β-MCA and T-MCA significantly increased. The most powerful elevation is UDCA rising by 3 fold. This indicates a less biliary toxicity after SLE supplementation. UDCA is the only BA approved by the U.S. Food and Drug Administration as a therapeutic agent for the treatment of primary biliary cirrhosis [47]. UDCA is known to have anti-oxidant, immune modulation, and anti-apoptotic properties [48]. A meta-analysis revealed that UDCA significantly reduced fasting glycemia, HbA1c, and insulin concentration [49]. Another systematic review summarized 12 randomized clinical trials testing UDCA therapy for NASH. Nine trials reported significant improvement of liver function including ALAT, ASAT, reduced steatosis, and fibrosis [50]. In our study, UDCA and HDL profile improvement observed after SLE supplementation is in accordance with previous published data of the lab in non-cirrhotic patients treated with UDCA during 2 years who presented a lower plasma cholesterol and a decreased large HDL2-to-small HDL3 ratio [51], suggesting a better liver metabolic function. This decrease in large HDL measured in the present study is accompanied by a lower amount of plasma ApoE concentration agreeing with the obligatory role for cholesterol and ApoE in the expansion of HDL [52]. Knowing that BA metabolism is also linked to gut microbiota, an effect of SLE on intestinal flora could be advanced. Indeed, it was reported that Spirulina protect against hepatic inflammation by modulating gut microbiota in aging [53].

## 5. Conclusions

In conclusion, these results indicate that oral spirulina liquid extract supplementation protects mice from hepatic fibrosis, inflammation, oxidative stress, and insulin resistance induced by a western diet concomitantly with the increased concentration of ursodeoxycholic acid.

## Figures and Tables

**Table 1:** Expression of key metabolic genes involved in non-alcoholic steatohepatitis (NASH). Values are expressed as relative values with the mean of control mice values set at 1. ** *p* < 0.01, *** *p* < 0.001 vs. Ctrl and ^$^ *p* < 0.05, ^$$^ *p* < 0.01, ^$$$^ *p* < 0.001 vs. western diet (WD) (*n* = 9–10/group).

Genes	Ctrl	WD	WDS1	WDS2
**Uptake**				
*Slc2a2*	1 ± 0.06	0.95 ± 0.04	0.79 ± 0.12	0.63 ± 0.10 ^$^
*Cd36*	1 ± 0.10	7.54 ± 0.7 ***	6..44 ± 0.96	4.3 ± 1.11 ^$^
**Lipogenesis**				
*Acc-1*	1 ± 0.06	1.05 ± 0.05	0.89 ± 0.14	0.65 ± 0.15 ^$^
*Scd-1*	1 ± 0.10	1.99 ± 0.14 ***	1.68 ± 0.22	1.15 ± 0.19 ^$$^
*Srebp-1*	1 ± 0.06	1.71 ± 0.11 **	1.30 ± 0.19	1.29 ± 0.17 *^p=^*^0.10^
*Dgat-1*	1 ± 0.04	1.13 ± 0.06	0.99 ± 0.09	0.89 ± 0.6 ^$^
**Oxidation**				
*Ppar-α*	1 ± 0.06	1.08 ± 0.05	0.93 ± 0.08	0.76 ± 0.05 ^$$^
*Cpt1a*	1 ± 0.03	2.09 ± 0.11 ***	1.89 ± 0.2	1.68 ± 0.18 *^p=^*^0.15^
**Fibrogenesis**				
*Col1a1*	1 ± 0.15	15.56 ± 0.87 ***	14.32 ± 1.06	8.10 ± 2.73 ^$$^
*Timp1*	1 ± 0.16	55.78 ± 3.25 ***	61.93 ± 7.40	27.99 ± 9.73 ^$^
**Inflammation**				
*Tgf-β1*	1 ± 0.10	2.12 ± 0.13 ***	1.99 ± 0.09	1.46 ± 0.19 ^$$^
*Tlr9*	1 ± 0.14	4.20 ± 0.37 ***	4.24 ± 0.37	2.10 ± 0.36 ^$$$^

Slc2a2: Solute carrier family 2 member 2; Cd36: Cluster of differentiation 36; Acc1: Acetyl-CoA carboxylase-1; Scd-1: Stearoyl-CoA desaturase-1; Srebp-1: Sterol regulatory element-binding protein-1; Dgat-1: Diacylglycerol O-acyltransferase-1; Ppar-α: Peroxisome proliferator-activated receptor α; Cpt1-a: Carnitine palmitoyltransferase1-a; Col1a1: Collagen type 1 α1; Timp1: Tissue inhibitor of metalloproteinase 1; Tgf-β1: Transforming growth factor β1; Tlr9: Toll-like receptor 9.

**Table 2:** Concentration of circulating lipoprotein cholesterol and apolipoprotein profiles. Cholesterol content in very low density lipoprotein (VLDL), low density lipoprotein (LDL) and high density lipoprotein (HDL). Plasma level of apolipoprotein (Apo) A–I, B100, E, C–II and C–III. *** *p* < 0.001 vs. Ctrl and ^$^ *p* < 0.05, ^$$^ *p* < 0.01, ^$$$^ *p* < 0.001 vs. WD (*n* = 9–10/group).

	Ctrl	WD	WDS1	WDS2
**Lipoproteins**				
VLDL-C (mg/dL)	3.9 ± 0.5	5.9 ± 0.3	3.3 ± 0.7 ^$^	1.8 ± 0.4 ^$$$^
LDL-C (mg/dL)	11.8 ± 0.3	57.2 ± 1.8 ***	47.0 ± 5.2	30.2 ± 5.1 ^$$$^
HDL1-C (mg/dL)	5.0 ± 0.3	50.1 ± 1.3 ***	41.5 ± 5.2	27.4 ± 5.2 ^$$$^
HDL2-C (mg/dL)	60.5 ± 3.1	160.8 ± 5.7 ***	138.5 ± 6.8	106.3 ± 25.2 ^$$$^
**Apolipoproteins**				
Apo A-I (mg/dL)	87.8 ± 8.6	946.7 ± 133.1 ***	915.8 ± 192.3	419.1 ± 103.3 ^$^
Apo B100 (mg/dL)	8.7 ± 1.0	19.9 ± 2.2 ***	17.9 ± 1.4	13.3 ± 2.4 ^$^
Apo E (mg/dL)	9.8 ± 0.4	60.0 ± 4.5 ***	53.3 ± 7.3	29.2 ± 6.6 ^$$^
Apo C-II (mg/dL)	0.7 ± 0.04	4.8 ± 0.3 ***	3.8 ± 0.3 ^$^	3.0 ± 0.3 ^$$$^
Apo C-III (mg/dL)	8.9 ± 1.0	23.0 ± 3.1 ***	15.6 ± 2.6	13.1 ± 2.1 ^$^

**Table 3:** Correlation between gallbladder UDCA content and biological variables in mice.

	Gallbladder UDCA (% Total BA)	
Variables	r	*p* Value
Body weight	−0.69	<0.0001
Fasting glycemia	−0.38	0.02
Fasting insulinemia	−0.49	0.003
AUC GTT	−0.45	0.006
ScAT weight	−0.65	<0.0001
Plasma total cholesterol	−0.72	<0.0001
Liver weight/Body weight ratio	−0.69	<0.0001
Fibrosis	−0.67	<0.0001
Steatosis	−0.64	<0.0001
Plasma ASAT	−0.59	0.0003
Plasma ALAT	−0.68	<0.0001
Liver O_2_^−^	−0.56	0.02
Liver NO	−0.60	0.005
Liver *Scd1* mRNA	−0.63	<0.0001
Liver *Tgfβ1* mRNA	−0.64	<0.0001
Liver *Col1a1* mRNA	−0.70	<0.0001
Liver *Timp1* mRNA	−0.65	<0.0001

AUC: Area under the curve; GTT: Glucose tolerance test; Scat: Subcutaneous adipose tissue; ASAT: Aspartate aminotransferase; ALAT: Alanine aminotransferase; O_2_^−^: superoxide anion; NO: nitric oxide; Scd-1: Stearoyl-CoA desaturase-1; Col1a1: Collagen type 1 α1; Timp1: Tissue inhibitor of metalloproteinase 1.

## Supplementary Materials

The following are available online at http://www.mdpi.com/2072-6643/11/1/194/s1, Figure S1: Drinking and food consumption reported to body weight, Figure S2: Anti-oxidative gene expression in oxidative tissues, Figure S3: Cholesterol metabolism-related gene expression, Table S1: Spirulina liquid extract (SLE) composition, Table S2: Composition of experimental diets, Table S3: Analytical parameters used for each proteotypic peptide in mouse, Table S4: Forward and reverse mouse primer sequences of genes used for real-time qPCR, Table S5: Correlation between gallbladder β-Muricholic acid (MCA) content and biological variables in mice.
