# Supplementary material for: Spirulina Liquid Extract Protects against Fibrosis Related to Non-Alcoholic Steatohepatitis and Increases Ursodeoxycholic Acid
Source: Nutrients. 2019 Jan 18;11(1):194. doi: 10.3390/nu11010194 (PMC6357008; doi:10.3390/nu11010194)
Supplement: Supplementary file 1 [file nutrients-11-00194-s001.pdf]

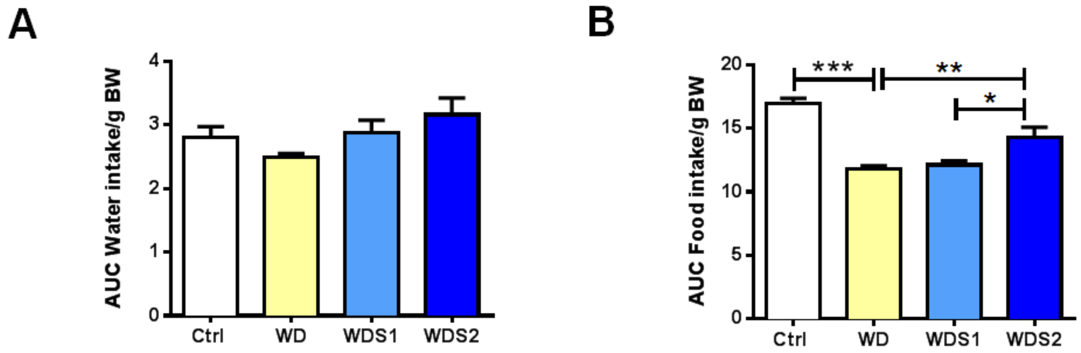

Figure S1. Drinking and food consumption reported to body weight. (**A**) Area under the curve (AUC) of water intake reported to body weight follow-up presented in Fig. 1E and (**B**) AUC of food intake reported to body weight follow-up presented in Fig. 1F. *$p < 0.05$, **$p < 0.01$, ***$p < 0.001$ (n = 9–10/group), ANOVA.

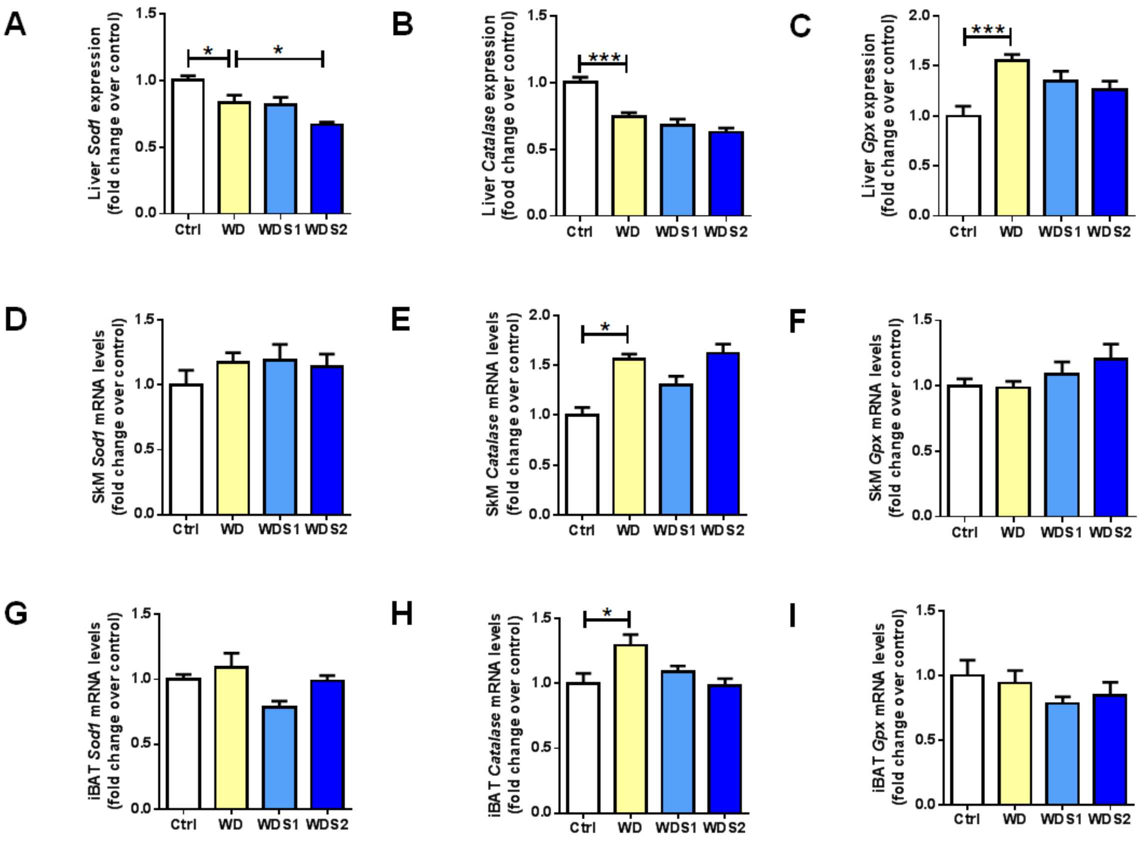

Figure S2. Anti-oxidative gene expression in oxidative tissues. (**A–C**) Anti-oxidative gene expression in liver, (**D–F**) skeletal muscle (SkM) and (**G–I**) interscapular brown adipose tissue (iBAT). *Sod1: superoxide dismutase 1; Gpx: glutathione peroxidase.* *$p < 0.05$, **$p < 0.01$, ***$p < 0.001$ (n = 5/group except n = 9–10/group for liver), ANOVA.

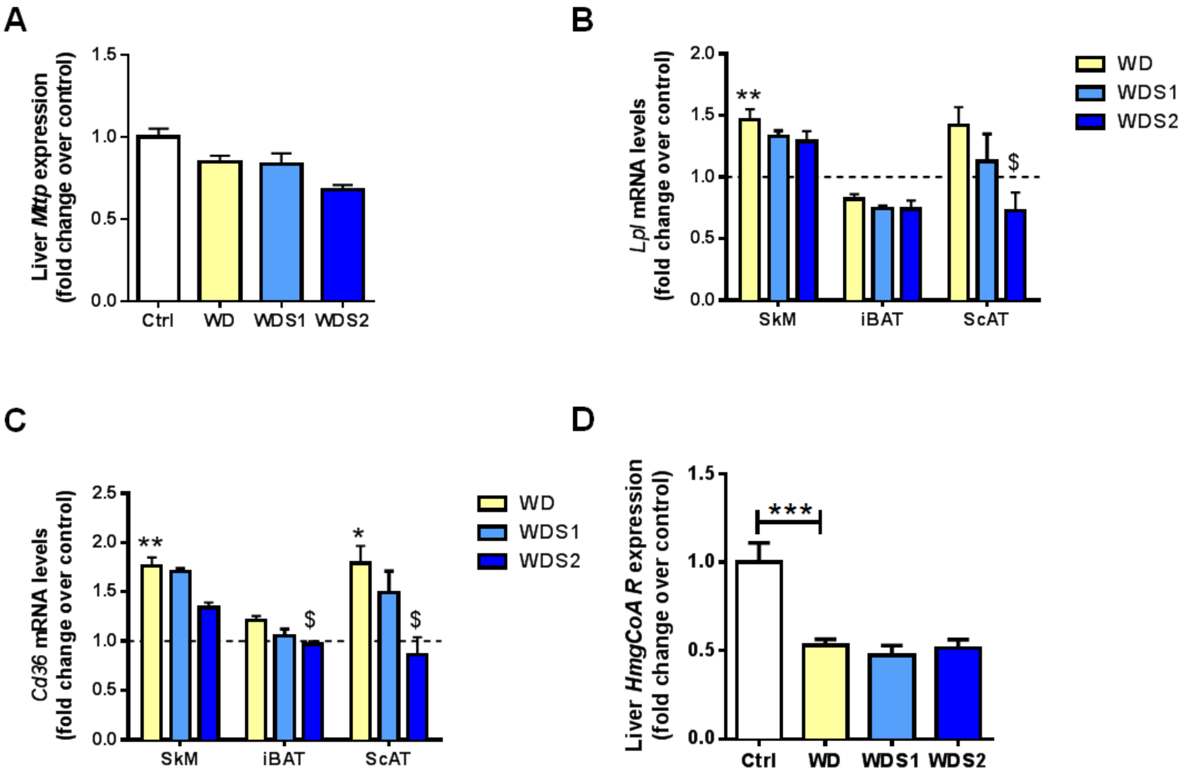

Figure S3. Cholesterol metabolism-related gene expression. (**A**) Liver expression of *Microsomal TG transfer protein* (*Mttp*). (**B**) *Lipoprotein lipase (Lpl)* and (**C**) *Cd36* gene expression in skeletal muscle (SkM), interscapular brown adipose tissue (iBAT) and subcutaneous adipose tissue (ScAT). (**D**) Hepatic *3-hydroxy-3-methyl-glutaryl-coenzyme A reductase (HMG-CoA R)* gene expression. $^{**}p < 0.01$, $^{***}p < 0.001$ *vs.* Ctrl and $^{\$}p < 0.05$ *vs.* WD ($n = 5$/group except $n = 9$–10/group for liver), ANOVA.

Table S1. Spirulina liquid extract (SLE) composition.

**Components SLE**	**Amount**
Phycocyanin (mg/L)	883
Energetic value (kJ/100g dry matter)	7
Total fat mass (g/100g)	Undetectable
Total carbohydrate (g/100g)	0.4
Amino acids (g/100g)	
Threonine	0.0090 (± 0.0013)
Aspartate	0.0187 (± 0.0026)
Alanine	0.0168 (± 0.0024)
Arginine	0.0138 (± 0.0019)
Leucine	0.0164 (± 0.0023)
Glutamate	0.0220 (± 0.0031)
Vitamin B12	Undetectable
Calcium (mg/kg)	330 (± 66)
Potassium (mg/kg)	55 (± 11)
Iron (mg/kg)	1.05 (± 0.42)
Copper (mg/kg)	0.2 (± 0.1)
Magnesium (mg/kg)	18 (± 3.6)

Table S2. Composition of experimental diets.

	**Chow diet**	**Western Diet**
**Energy value (Atwater, MJ/kg)**	13.97	19.95
**Proteins (%)**	16.1	17.1
**Lipids (%)**	3.1	23.2
Saturated fatty acids (%)	0.6	16.8
Insaturated fatty acids (%)	2.5	6.4
Total omega 3 fatty acids (%)	0.2	0.1
Total omega 6 fatty acids (%)	1.6	1.0
Total trans fatty acids (%)	0.003	0.5
**Carbohydrates (%)**	60.4	49.9
**Fibers (%)**	3.9	3.5
**Minerals (%)**	4.6	3.1
**Cholesterol (%)**	0	2

Table S3. Analytical parameters used for each proteotypic peptide in mouse.

Protein (MW)	Name	Peptide	Fragment	Cone/collision (V)	MRM (*m*/*z*)
ApoA-I (27.95 kDa)	M0	ARPALEDLR	y_7^+	30/28	521.0 → 813.6
	IS	ARPALEDL-[$^{13}C_6{}^{15}N_4$]R			526.0 → 823.6
ApoB100 (506.37 kDa)	M0	DFSIWEETGLK	y_7^+	30/22	663.2 → 862.5
	IS	DFSIWEETGL-[$^{13}C_6{}^{15}N_2$]K			667.2 → 870.5
ApoC-II (8.30 kDa)	M0	TYPISMDEK	y_7^+	30/18	542.5 → 819.5
	IS	TYPISMDE-[$^{13}C_6{}^{15}N_2$]K			546.5 → 827.5
ApoC-III (8.89 kDa)	M0	GWMDNHFR	y_6^+	35/25	532.0 → 819.5
	IS	GWMDNHF-[$^{13}C_6{}^{15}N_4$]R			537.0 → 829.5
ApoE (33.97 kDa)	M0	LGPLVEQGR	y_5^+	25/30	484.8 → 588.3
	IS	LGPLVEQG-[$^{13}C_6{}^{15}N_4$]R			489.8 → 598.3

M0, unlabeled peptide; IS, internal standard; MRM, multiple reaction monitoring.

Table S4. Forward and reverse mouse primer sequences of genes used for real-time qPCR.

Gene symbol	Forward 5′ to 3′	Reverse 5′ to 3′
Acc1	TTC TGA ATG TGG CTA TCA AGA CTG A	TGC TGG GTG AAC TCT CTG AAC A
Catalase	CCA GCG ACC AGA TGA AGC AG	CCA CTC TCT CAG GAA TCC GC
Cd36	GTT AAA CAA AGA GGT CCT TAC ACA TAC AG	CAG TGA AGG CTC AAA GAT GGC
Col1a1	CTC CTG GCA AGA ATG GAG AT	AAT CCA CGA GCA CCC TGA
Cpt1a	GAA GAA GAA GTT CAT CCG ATT CAA G	GAT ATC ACA CCC ACC ACC ACG
Dgat-1	ATC CAG ACA ACC TGA CCT ACC G	AGA ACT CGT CGT AGC AGA AAG C
Gpx	TTC GGA CAC CAG GAG AAT GG	TAA AGA GCG GGT GAG CCT TC
HMGCoA R	CTT GTG GAA TGC CTT GTG ATT G	GAA GAA TGT CAT GAA CAC AAA GTA GTT G
Lpl	CAA GGT CAG AGC CAA GAG AAG C	GTT GCT TGC CAT CCT CAG TCC
Mttp	TGA GCG GCT ATA CAA GCT CA	CTG GAA GAT GCT CTT CTC GC
Pparα	CGT TTG TGG CTG GTC AAG TTC G	AGT GGG GAG AGA GGA CAG ATG G
Scd-1	GCT CTA CAC CTG CCT CTT CGG	CCG TGC CTT GTA AGT TCT GTG G
Slc2a2	GTC CAG AAA GCC CCA GAT ACC	GTG ACA TCC TCA GTT CCT CTT AG
Sod1	CAG CAT GGG TTC CAC GTC CA	CAC ATT GGC CAC ACC GTC CT
Srebp-1	TCC TGC CTC CGA GCT TCC C	TGG TGG CTG CTG AGT GTT TCC
Tbp	ACT TCG TGC AAG AAA TGC TGA A	GCA GTT GTC CGT GGC TCT CT
Tgf-β1	TGG AGC AAC ATG TGG AAC TC	CAG CAG CCG GTT ACC AAG
Timp1	GCA AAG AGC TTT CTC AAA GAC C	AGG GAT AGA TAA ACA GGG AAA CAC T
Tlr9	GGG CCC ATT GTG ATG AAC C	GCT GCC ACA CTT CAC ACC AT

Acc1: Acetyl-CoA carboxylase-1; Cd36: Cluster of differentiation 36; Col1a1: Collagen type 1 α1, Cpt1-a: Carnitine palmitoyltransferase1-a; Dgat-1: Diacylglycerol O-acyltransferase-1; Gpx: Glutathione peroxidase; HMGCoA-R: 3-hydroxy-3-methyl-glutaryl-coenzyme A reductase; Lpl: Lipoprotein lipase; Mttp: Microsomal triglyceride transfer protein; Ppar-α: Peroxisome proliferator-activated receptor α; Scd-1: Stearoyl-CoA desaturase-1; Slc2a2: Solute carrier family 2 member 2; Sod1: Superoxide dismutase 1; Srebp-1: Sterol regulatory element-binding protein-1; Tbp: TATA-box binding protein; Tgf-β1: Transforming growth factor β1; Timp1: Tissue inhibitor of metalloproteinase 1, Tlr9: Toll-like receptor 9.

Table S5. Correlation between gallbladder β-Muricholic acid (MCA) content and biological variables in mice.

	**Gallbladder β-MCA (% Total BA)**	
**Variables**	**r**	**p value**
Body weight	−0.63	<0.0001
Fasting glycemia	−0.24	0.16
Fasting insulinemia	−0.47	0.004
AUC GTT	−0.42	0.01
ScAT weight	−0.62	<0.0001
Plasma total cholesterol	−0.71	< 0.0001
Liver weight/Body weight ratio	−0.69	< 0.0001
Fibrosis	−0.63	< 0.0001
Steatosis	−0.58	0.0002
Plasma ASAT	−0.52	0.002
Plasma ALAT	−0.64	< 0.0001
Liver O_2^-	−0.43	0.06
Liver NO	−0.42	0.06
Liver *Scd1* mRNA	−0.55	0.0005
Liver *Tgfβ1* mRNA	−0.57	0.0003
Liver *Col1a1* mRNA	−0.67	<0.0001
Liver *Timp1* mRNA	−0.65	<0.0001

AUC: Area under the curve; GTT: Glucose tolerance test; Scat: Subcutaneous adipose tissue; ASAT: Aspartate aminotransferase; ALAT: Alanine aminotransferase; O_2^-: superoxide anion; NO: nitric oxide; Scd-1: Stearoyl-CoA desaturase-1; Col1a1: Collagen type 1 α1; Timp1: Tissue inhibitor of metalloproteinase 1.
